# A rare regulatory variant in the *MEF2D* gene affects gene regulation and splicing and is associated with a SLE sub-phenotype in Swedish cohorts

**DOI:** 10.1038/s41431-018-0297-x

**Published:** 2018-11-20

**Authors:** Fabiana H. G. Farias, Johanna Dahlqvist, Sergey V. Kozyrev, Dag Leonard, Maria Wilbe, Sergei N. Abramov, Andrei Alexsson, Gerli R. Pielberg, Helene Hansson-Hamlin, Göran Andersson, Karolina Tandre, Anders A. Bengtsson, Christopher Sjöwall, Elisabet Svenungsson, Iva Gunnarsson, Solbritt Rantapää-Dahlqvist, Ann-Christine Syvänen, Johanna K. Sandling, Maija-Leena Eloranta, Lars Rönnblom, Kerstin Lindblad-Toh

**Affiliations:** 1 Science for Life Laboratory, Department of Medical Biochemistry and Microbiology, Uppsala University, Box 582, SE-751 24 Uppsala, Sweden; 2 Science for Life Laboratory, Department of Medical Sciences, Section of Rheumatology, Uppsala University, SE-751 85 Uppsala, Sweden; 3 Department of Animal Breeding and Genetics, Swedish University of Agricultural Sciences (SLU), Box 7023, SE-750 07 Uppsala, Sweden; 4 Institute of Fundamental Medicine and Biology, Kazan Federal University, Kazan, 420008 Russia; 5 Department of Clinical Sciences, Swedish University of Agricultural Sciences (SLU), Box 7054, SE-750 07 Uppsala, Sweden; 6 Department of Clinical Sciences Lund, Lund University, Skane University Hospital, SE-221 00 Lund, Sweden; 7 Department of Clinical and Experimental Medicine, Rheumatology/Division of Neuro and Inflammation Sciences, Linköping University, SE-581 85 Linköping, Sweden; 8 Rheumatology Unit, Department of Medicine, Solna, Karolinska Institutet, Karolinska University Hospital, SE-171 76 Stockholm, Sweden; 9 Department of Public Health and Clinical Medicine/Rheumatology, Umeå University, SE-901 85 Umeå, Sweden; 10 Department of Medical Sciences, Molecular Medicine and Science for Life Laboratory, Uppsala University, SE-754 11 Uppsala, Sweden; 11 Broad Institute, Cambridge, 7 Cambridge Center, Cambridge, MA 02142 USA; 12 Present Address: Department of Psychiatry, Washington University School of Medicine, St. Louis, MO 63110 USA; 13 Present Address: Science for Life Laboratory, Department of Immunology, Genetics and Pathology, Uppsala University, Box 582, SE-751 24 Uppsala, Sweden

**Keywords:** Gene regulation, Sequencing

## Abstract

Systemic lupus erythematosus (SLE) is an autoimmune disorder with heterogeneous clinical presentation and complex etiology involving the interplay between genetic, epigenetic, environmental and hormonal factors. Many common SNPs identified by genome wide-association studies (GWAS) explain only a small part of the disease heritability suggesting the contribution from rare genetic variants, undetectable in GWAS, and complex epistatic interactions. Using targeted re-sequencing of coding and conserved regulatory regions within and around 215 candidate genes selected on the basis of their known role in autoimmunity and genes associated with canine immune-mediated diseases, we identified a rare regulatory variant rs200395694:G > T located in intron 4 of the *MEF2D* gene encoding the myocyte-specific enhancer factor 2D transcription factor and associated with SLE in Swedish cohorts (504 SLE patients and 839 healthy controls, *p* = 0.014, CI = 1.1–10). Fisher’s exact test revealed an association between the genetic variant and a triad of disease manifestations including Raynaud, anti-U1-ribonucleoprotein (anti-RNP), and anti-Smith (anti-Sm) antibodies (*p* = 0.00037) among the patients. The DNA-binding activity of the allele was further studied by EMSA, reporter assays, and minigenes. The region has properties of an active cell-specific enhancer, differentially affected by the alleles of rs200395694:G > T. In addition, the risk allele exerts an inhibitory effect on the splicing of the alternative tissue-specific isoform, and thus may modify the target gene set regulated by this isoform. These findings emphasize the potential of dissecting traits of complex diseases and correlating them with rare risk alleles with strong biological effects.

## Introduction

Systemic lupus erythematosus (SLE) is a chronic inflammatory autoimmune disease that predominantly affects women of childbearing age [1]. A number of studies exploring the genetic basis of SLE in diverse populations identified over 40 risk loci [2], however it was estimated that these loci explain only about 30% of SLE heritability [3], indicating that disease pathogenesis results from a combined effect of different mechanisms and even a larger number of genes. The recently proposed omnigenic model of complex traits suggests that virtually any gene with regulatory variants active in relevant tissue may contribute to disease pathogenesis [4]. Genes are highly interconnected within the cell-specific gene networks, and thus any effect on one gene with regulatory function, that is not even directly related to disease pathways, would lead to waves of perturbations in other genes that would result in increased disease susceptibility. This implies that many non-canonical susceptibility genes remain to be discovered.

Apart from humans, SLE-like disorders have been studied in mice [5] and dogs [6]. These studies revealed potentially shared disease mechanisms across species. We have previously identified five loci associated with a SLE-related disease complex in dogs [7], leading to the detection of a risk haplotype that affects the expression of the *BANK1* gene [8], which is also associated with human SLE [9] and cause perturbations in B-cell signaling pathways in mice [10]. Identification of shared mechanisms and genes between human and animal diseases could further improve our understanding of SLE.

While GWAS lacks the capability of identifying rare genetic variants, next generation sequencing provides better resolution for discovery of such variants. Here, we used targeted enrichment and high-throughput sequencing of genes from pathways relevant for human immunity, SLE-associated genes, and genes associated with the canine SLE-related disease. Our approach revealed an association of a rare regulatory variant with SLE in the Swedish population.

## Materials and methods

### Cases and controls

To detect novel disease-associated rare variants, we performed targeted re-sequencing of 17 healthy controls and 156 patients; 16 of these had medical record data indicating that they or their parents were born in another country than Sweden (the remaining hereafter referred to as “Swedish”). All patients fulfilled four or more of the American College of Rheumatology (ACR) classification criteria for SLE [11, 12] and were enrolled at the outpatient rheumatology clinic at Uppsala University Hospital, Sweden. Clinical data, including age, sex, disease duration, smoking habits, information of ACR, and systemic lupus international collaborating clinics (SLICC) classification criteria [13, 14], SLICC/ACR damage index (SLICC DI), major cardiovascular event (MCE; myocardial infarction, stroke or transient ischemic attack) and Raynaud, as well as results of autoantibody analyses, were collected from medical records. A summary of patient characteristics is presented in Supplementary Table 1. An additional cohort of 364 Swedish SLE cases (average age = 51, 84% females) was used for variant validation by genotyping and for further genetic analysis. A total of 837 healthy blood donors from Uppsala Bioresource (Uppsala, Sweden) matched for age and sex (average age = 50, 88% females) were used as controls. All patients and controls gave their informed consent. The regional ethics committee at Uppsala, Sweden approved the study protocols EPN Uppsala Dnr 00–227 and Dnr 2016/155.

### Gene array capture and sequencing data analysis

For all 215 genes (Supplementary Table 2) chosen for targeted re-sequencing, the following regions were included: all annotated coding exons, 5′ UTRs, 3′ UTRs and all conserved elements with a SiPhy [15] lodscore of > 7.5 based on 29 mammals alignment [16], and located within 100 kb 5′ and 3′ of the genes as well as introns. The tiling array comprised 5,059,619 bp in total. Detailed description of array design and sequence analysis is in Supplementary Methods. DNA samples from patients were allocated into ten pools (Supplementary Table 3) and DNA from 17 healthy controls was pooled together. Paired-end sequencing was performed using Illumina HighSeq2000 at the SNP&SEQ Technology Platform (National Genomics infrastructure, SciLifeLab, Uppsala, Sweden), yielding 100 bp reads. Sequence data was submitted to European Nucleotide Archive (ENA) http://www.ebi.ac.uk/ena/data/view/PRJEB8904 (study accession number: PRJEB8904).

### Variant validation and genetic analysis

**Fig. 1:**
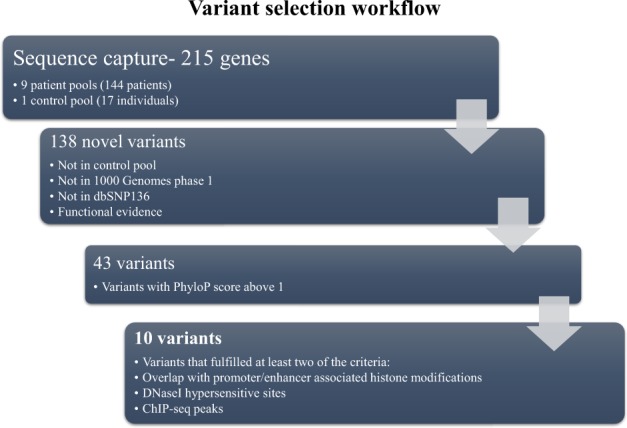
Flowchart of variant selection. Variant selection was based on a series of filters to remove variants without enough evidence for regulatory potential

Variants were selected based on a series of filters with strict cutoff thresholds and functional evidence based on ENCODE data and Phylo P scores (Fig. 1). Genotyping by pyrosequencing was used to validate candidate SNPs (*n* = 10) and to identify individuals carrying the variants in the primary patient cohort and 96 Swedish healthy blood donors. Genotyping of the additional cases and controls was performed either by ABI TaqMan allelic discrimination assay on the ABI 7900HT system (Applied Biosystems) or Sanger sequencing. Fisher’s exact test was used to analyze associations between 3 candidate SNPs and disease status. The three associated variants were tested for HWE: rs200395694:G > T *p*-value 0.91; rs867059436:G > A *p*-value 0.98; rs576275580:G > A *p*-value 0.88.

### Reporter assays and EMSA

The allelic effects were studied using electrophoretic mobility shift assays (EMSA) and luciferase reporter assays. For detailed description see Supplementary methods.

### Minigenes and splicing analysis

The *MEF2D* minigene constructs containing different allelic variants of rs200395694 were prepared as follows: one intact 2.9 kb region (positions: chr1:156,479,713–156,482,610; hg38) containing four exons including two alternative exons α1 and α2 [17] together with introns was amplified by PCR from a DNA sample with known heterozygous genotype and cloned into pcDNA3.1 D/V5-His -TOPO vector (Invitrogen). The resulting plasmids were validated by Sanger sequencing and purified with EndoFree Plasmid Maxi kit (Qiagen) for transfection experiments. The minigenes were tested in Jurkat, THP-1, HEK293, and C2C12 cell lines using quantitative reverse transcription (RT-PCR) for transcript detection (Supplementary Methods**)**.

### Statistical analyses of SLE phenotypes and candidate SNP

The complete clinical data were available for 140 Swedish patients and these were used for analysis of associations between 21 SLE phenotypes using Fisher’s exact test (binary variables) and Mann–Whitney *U*-test (non-binary and ordinal variables). The ACR criterion 10 was removed from genotype-phenotype association analyses as it was well represented by the autoantibodies. Statistical analyses were performed using R. The *p*-value threshold after Bonferroni correction for 21 tests is 2.4 × 10^−3^.

## Results

### Targeted re-sequencing and variant selection

In order to find novel rare variants relevant to SLE pathogenesis, we performed targeted re-sequencing of 215 candidate genes and their potentially regulatory regions including elements highly conserved across mammals [16]. The list of genes comprises known human SLE-associated genes and genes involved in immune response and autoimmunity (*n* = 77), the nuclear factor of activated T cells (NFAT) pathway genes (*n* = 98), genes in regions associated with dog SLE-like disease [7] and other dog immune-mediated diseases (*n* = 40) (Supplementary Table 2).

We successfully re-sequenced the candidate regions in 140 SLE patients (nine pools) and 17 Swedish healthy individuals (one pool). One of the patient pools containing 4 Swedish and 12 non-Swedish SLE samples failed library preparation and was not sequenced. The average coverage was 3775 X per pool. A summary of the sequencing results can be found in Supplementary Table 3.

A total of 14,206 SNPs were identified in the case pools that were absent in the control pool. To identify novel case-only variants, all SNPs found only in the case pools were also compared against the 1000 Genomes database (1000G phase 1) and dbSNP136 [18] and only novel SNPs were kept. Later, however, our key SNP was found in dbSNP137. A series of filters with strict cutoff thresholds (Fig. 1) were further applied to select variants with the most evidence for potential regulatory function. Ten SNPs located in six genes fulfilled our criteria by combining strong signals for regulatory potential and were kept for further validation (Supplementary Table 4).

### Genotyping and genetic analysis of candidate variants

Since the variant discovery was performed on pooled DNA, we genotyped our patient cohort in order to identify the individuals carrying the alternative alleles of the ten selected SNPs. In addition, a small control group of 96 Swedish healthy blood donors was also genotyped for comparison. After genotyping, we excluded seven SNPs based on their occurrence in the control group or presence in only one patient. The SNPs that remained were the following: rs200395694:G > T (hg19 chr1:g.156450591 G > T), located in the myocyte enhancer factor 2D (*MEF2D)* gene, rs867059436:G > A (hg19 chr14:g.22958952 G > A), located in the T-cell receptor alpha (*TCRA*) gene locus, and rs576275580:G > A (hg19 chr15:g.89437973 G > A) located in the first intron of the hyaluronan and proteoglycan link protein 3 (*HAPLN3)* gene.

The three SNPs were further genotyped in an additional set of 364 Swedish SLE cases and 741 control samples and used for genetic association analysis. Combining all genotyped cases and controls we observed a significant association with SLE only for the *MEF2D* variant rs200395694 (*p* = 0.014). There were in total 12 heterozygotes out of 504 patients and 6 heterozygotes out of 839 controls giving an allele frequency of 0.011 and 0.003, respectively. The SNP is present in an updated version of dbSNP, and the allele frequency in 1000G project is 0.001. The allele frequency in healthy controls observed in our study is similar to those reported for the general Swedish population, according to SweFreq (MAF 0.002) (https://swegen-exac.nbis.se/) [19]. Only two alternative alleles were detected in the Swedish population. Of note, there are only heterozygotes reported in both 1000G and SweFreq, and not a single homozygote was identified for the alternative allele. The other two SNPs were not associated in the larger cohort (*HAPLN3:* *p* = 0.818; *TCRA:* *p* = 0.188, Supplementary Table 5).

### Functional effects of candidate variant

**Fig. 2:**
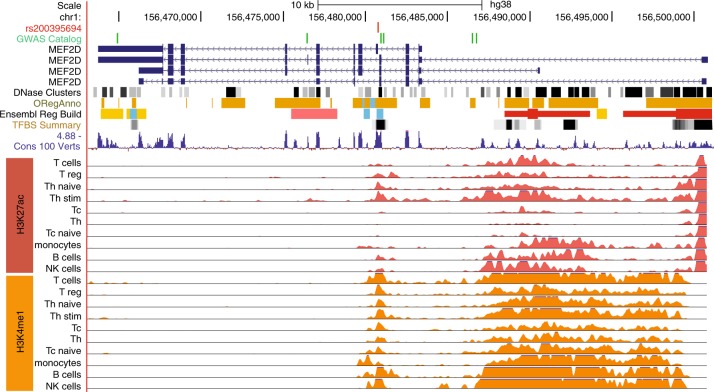
Functional annotation of the *MEF2D* region with SNP rs200395694. The variant is located in intron 4 of the *MEF2D* gene and overlaps with strong regulatory marks including DNase I hypersensitivity region (DNase Clusters), open regulatory region associated with active gene expression (ORegAnno), conserved transcription factor binding sites (TFBS Summary) and region conservation across 100 vertebrates (Cons 100 Verts) (http://genome.ucsc.edu/). The histone modification marks (H3K27ac, H3K4me1) associated with active enhancers mapped for blood cell populations according to Roadmap Epigenomics (http://egg2.wustl.edu/roadmap/web_portal/) indicate the presence of a cell type-specific enhancer. The known GWA signals located in the *MEF2D* gene region for migraine and blood cell traits are shown by green vertical lines (GWAS Catalog) [31–33]

The associated SNP rs200395694 is located in the small intron 4 of the myocyte enhancer factor 2D (*MEF2D)* gene (Ensembl:ENSG00000116604) (encoded on the negative strand: ref C, alt A) and overlaps with a DNase I hypersensitive site, histone modifications and transcription factor binding sites (Fig. 2). The PhyloP score of 4.2 indicates high conservation among mammals and two regulatory motifs for Elk1 and GABP transcription factors overlap with the variant (http://genome.ucsc.edu/). The region also contains chromatin modification marks for enhancers active in T, B, NK cells, and monocytes (Fig. 2) (http://www.roadmapepigenomics.org) [20]. The Regulome database annotates the variant as likely to affecting binding with the score 2a (http://regulomedb.org/snp/chr1/156450590) [21]. Further analyses using TRANSFAC [22] and TRAP [23], indicated stronger binding affinities to the alternative allele for several transcription factors (Supplementary Table 6).

**Fig. 3:**
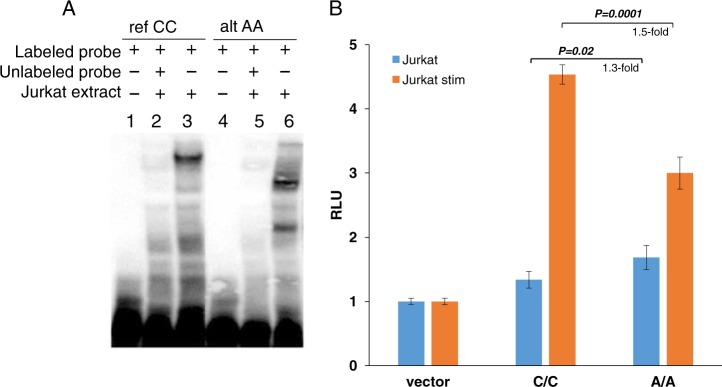
Binding and regulatory potential of rs200395694 alleles. **a** EMSA results with nuclear extract from Jurkat cells. **b** Luciferase reporter assay performed in Jurkat. Bars represent mean values ± SD. RLU relative light units. Statistical analysis was done using an unpaired *t-*test

The potential for protein-binding of rs200395694 was first investigated by EMSA using nuclear extracts from Jurkat T-cell line to confirm the Roadmap T-cell-specific enhancer activity and tested also in Daudi B-cell line. We observed differential protein-binding between the reference and the alternative allele in nuclear extracts from both cell lines (illustrated with Jurkat T cells; Fig. 3a). Next, we explored the regulatory effect of the SNP by luciferase reporter assays. The opposite effect of the alternative allele A on the reporter gene expression in different cells and under different conditions was detected. Thus, the alternative A allele induced expression in non-stimulated Jurkat (1.3-fold, *p* *=* *0.02*) and K562 cells (1.8-fold, *p* *=* *0.0001*) compared to the reference C allele (Fig. 3b, Supplementary Fig. 1), while in THP-1 this allele suppressed the reporter, and no significant allelic difference was seen in HeLa and Daudi. Interestingly, upon Jurkat stimulation, the reference allele showed much higher luciferase activity (1.5-fold, *p* *=* *0.0001*), suggesting active regulation by another transcription factor(s) with increased binding affinity to the C allele. In stimulated K562, the luciferase expression driven by the risk allele A remains higher compared to the reference allele C (1.4-fold, *p* *=* *0.0001*) (Supplementary Fig. 1).

**Fig. 4:**
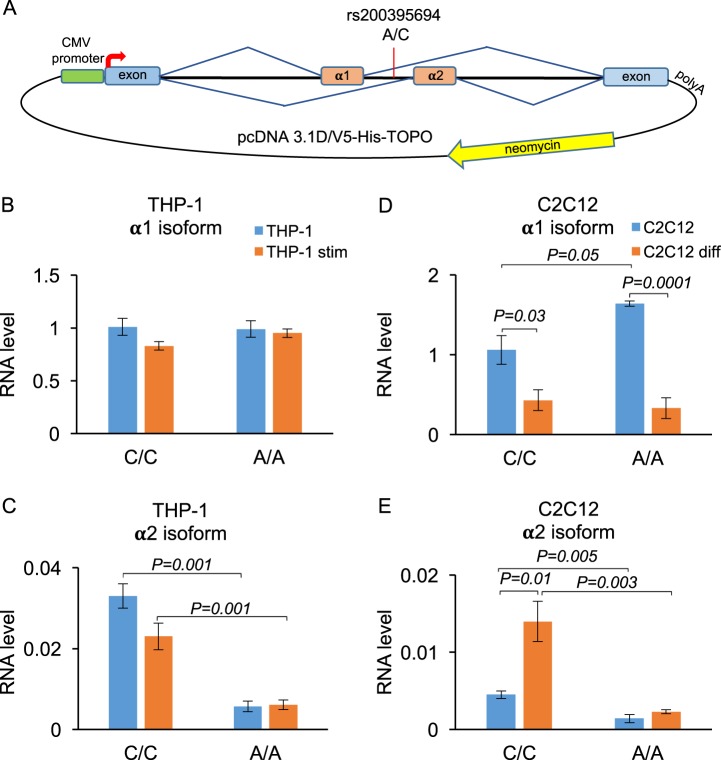
Analysis of alternative splicing with minigenes. **a** Minigenes with alternative alleles were cloned into pcDNA3.1 vector between the CMV promoter and the polyadenylation site. Neomycin gene was used for transfection normalization. **b**–**e** Levels of alternative isoforms transcribed from minigenes transfected into THP-1 cells (**b**, **c**) and C2C12 cells (**d**, **e**) were measured by quantitative RT-PCR. THP-1 cells were stimulated with 100 ng/ml of LPS and 10 ng/ml of interferon gamma for 12 h. C2C12 cells were differentiated with 2% horse serum for 64 h. Bars represent mean values ± SEM

Given the reported alternative tissue-specific splicing of the exon located 22 bp downstream of rs200395694 [17, 24] we also studied the potential effect of the rare allele A on splicing using minigenes (Fig. 4a). Both isoforms are expressed in human peripheral blood mononuclear cells (PBMC), monocytic cells THP-1, myelogenous leukemia cells K562, B-cell line Daudi, and murine myoblasts C2C12, but only the major α1 transcript was detected in Jurkat, cervical cancer cells HeLa and human embryonic kidney cells HEK293 (Supplementary Fig. 2). The minigene constructs with different genotypes were transfected into two cell lines, not expressing the α2 isoform, Jurkat and HEK293, and two lines with detected expression of α2, THP-1, and C2C12 cells. The latter cell line was chosen as a control, since the splicing of the *MEF2D* isoforms has previously been thoroughly examined in C2C12 cells [17]. No allelic difference was detected for the α1 isoform transcribed from the minigenes in THP-1, HEK293 and Jurkat, and only a marginally significant increase was detected in the A allele-containing constructs transfected in C2C12 cells (Fig. 4b–e and Supplementary Fig. 3). The alternative isoform α2 generated from the minigene with the rare A allele was significantly repressed in all tested cells. Both isoforms were downregulated upon stimulation of Jurkat and THP-1, while in C2C12 the major α1 isoform was also downregulated upon cell differentiation, and the minor α2 isoform was upregulated in the reference allele and remained inhibited in the rare A allele (Fig. 4). The downregulation of α1 and induction of α2 upon cell C2C12 differentiation is in agreement with the previously reported results [17].

### Association analysis of the candidate variant with clinical manifestations

Finally, we investigated whether the rs200395694 variant was specifically associated with any of the clinical phenotypes within the SLE cohort (140 SLE Swedish patients from the discovery set for which we have close to complete clinical information). The clinical characteristics for all patients and for the patients carrying the alternative allele for the candidate SNP are presented in Table 1. We observed a nominally significant association between the *MEF2D* rs200395694-A allele and presence of anti-Sm antibodies (*p* = 0.0058) and anti-RNP antibodies (*p* = 0.017) with Fisher’s exact test. Previous autoantibody profile analyses have shown that anti-RNP and anti-Sm antibodies cluster together in SLE cohorts and that anti-RNP antibodies are associated with Raynaud’s phenomenon [25]. In our data, all three individuals carrying the *MEF2D* rs200395694-A allele were positive for these three phenotypes, as compared to 11 individuals (8.5%) of all the patients, with a significant association between this triad of disease manifestations and the rs200395694-A allele (*p* = 0.00037; Fisher’s exact test).

**Table 1:** Clinical data of Scandinavian patients and patients carrying candidate SNPs in *MEF2D*

	Swedish patients (140)	*MEF2D* rs200395694-A (*n* = 3)	*p*-value
Females/males	125/15	2/1	
Age, mean (range)	48 (20–85)	47 (39–51)	
Disease duration, mean (range)	17 (0–63)	17 (8–27)	
Smoking (ever), no. (%)	50 (36)	2 (67)	
ACR criteria (1982), no. (%)			
1. Malar rash	91 (65)	2 (67)	1
2. Discoid rash	43 (31)	1 (33)	1
3. Photosensitivity	97 (69)	2 (67)	1
4. Oral ulcers	41 (29)	1 (33)	1
5. Arthritis	101 (72)	3 (100)	0.56
6. Serositis	50 (36)	2 (67)	1
7. Renal disorder	33 (24)	1 (33)	0.557
8. Neurologic disorder	7 (5)	0	1
9. Hematologic disorder	87 (62)	3 (100)	0.289
10. Immunologic disorder	87 (62)	3 (100)	-
11. Anti-nuclear antibodies	137 (98)	3 (100)	1
Total ACR criteria, median (range)	5 (4–9)	7 (5–8)	0.179
SLICC DI, median (range)	0 (0–6)	2 (0–3)	0.202
Presence of, no. (%)			
Anti-dsDNA antibodies	82 (59)	3 (100)	0.267
Anti-Sm antibodies	26 (19)	3 (100)	0.0058
Anti-RNP antibodies	37 (26)	3 (100)	0.017
Anti-SSA antibodies	67 (48)	1 (33)	1
Anti-SSB antibodies	31 (22)	2 (67)	0.123
Anti-cardiolipin antibodies (IgM or IgG)	52 (39)	1 (33)	1
Major cardiovascular event	16 (11)	1 (33)	0.307
Raynaud	64 (50)	3 (100)	0.244
Raynaud, anti-RNP and anti-Sm antibodies	11 (8.7)	3 (100)	0.00037

*p*-values were calculated using Fisher’s exact test and Mann–Whitney *U* -test. ACR criterion 10 (Immunologic disorder) was excluded from the association analyses as it was strongly correlated with presence of anti-dsDNA, anti-RNP, and anti-Sm. Missing data (*n*) for Raynaud: 13; Anti-cardiolipin antibodies: 5; Smoking: 1; remaining parameters: 0

*ACR* American College of Rheumatology, *SLICC*  Systemic Lupus International Collaborating Clinics, *SLICC DI*  SLICC damage index, *Age*  age at time of data collection, *Major cardiovascular event*  transient ischemic attack, stroke, or myocardial infarction

## Discussion

SLE is considered as the prototype of complex autoimmune diseases with significant contribution from genetic background. In an attempt to map loci associated with the disease, numerous GWA studies have been performed in diverse populations (reviewed in ref. [2]). The main drawback of such studies is the inability to detect genetic effects from rare and low frequency variants that may have direct causal effects on genes. While whole-genome sequencing is still a demanding experiment, the use of targeted sequencing of specific regulatory regions in selected genes could be a strategy of choice when analyzing large patient cohorts. As a proof of principle, we focused on coding and conserved regulatory regions in 215 genes selected for their role in immune pathways or for being previously associated with SLE in humans or dogs.

The power to detect rare disease-associated variants depends on the ability to study specifically variants that are likely to have functional consequence. At present, it is much easier to assign potential detrimental function to coding variants. However, based on common variants associated with common diseases (majority of GWA studies signals reside outside coding regions [26]), it is likely that also non-coding variants are involved in the disease pathogenesis and that they can have both low and high effect sizes. For example, for a scenario where selection is relatively weak (*s* = 10^−3^) we would need 260 cases to detect an allele with and odds ratio of 20, while a variant with a twofold increased risk would require 28,000 cases [27]. In this study, we attempted to hedge our bets for discovery of novel rare variants affecting SLE in multiple ways, we: (1) targeted genes in pathways associated to SLE in humans or dogs, (2) targeted evolutionarily conserved non-coding elements in addition to coding regions of these genes, (3) we used multiple functional genomics data sets to point out variants with candidate functions, and (4) looked for novel variants not present in controls and available databases, thus increasing the likelihood that the detected variants would be detrimental. Based on this we were able to find one variant that was both functional and associated to disease sub-phenotypes. However, it is worth realizing that the current data set will include a lot of false negatives.

We identified a rare variant located in intron 4 of *MEF2D* gene significantly enriched in Swedish SLE patients. This gene was selected for targeted sequencing based on its role in the NFAT pathway. The genetic association signals in the NFAT pathway genes with SLE were identified in dogs [7], thereby making canine associated genes and pathways valuable candidates for investigation in human disease. *MEF2D* encodes for a member of the myocyte enhancer factor 2 family of transcription factors, widely expressed in different tissues, including all hematopoietic cell populations [28]. MEF2D is an essential transcriptional activator of the interleukin 2 (*IL-2*) gene [29]. The dysregulation of IL-2 production is a common characteristic of T cells in SLE [30]. Interestingly, several common SNPs in the *MEF2D* gene have been reported to be strongly associated with migraine [31, 32] and blood cell phenotypes [33], but none of them have been implicated in association with any autoimmune disease.

The A allele of the *MEF2D* SNP rs200395694 was also significantly associated with the triad of disease manifestations comprising of anti-Sm, anti-RNP antibodies, and Raynaud’s phenomenon in our patient cohort. The association between anti-RNP and anti-Sm antibodies to Raynaud’s phenomenon has been previously established [25], implying a common mechanism involved in the etiology of these manifestations; nonetheless to our knowledge, no gene has been associated with this combined phenotype to date.

The combined evidence from genomic annotations suggested the presence of an active cell type-specific enhancer in the region harboring the SNP. This is fully supported by the results obtained in our in vitro experiments, where they indicate that both alleles bind specifically to protein complexes, but that these are different for the two alleles. This suggests that different transcription factors may recognize the fragment depending on the allele present and eventually participate in gene regulation (Fig. 3a). The luciferase reporter assay confirmed the cell line-specific enhancer with significant differential allelic effect: the two alleles had different levels of expression for both unstimulated and stimulated Jurkat cells (Fig. 3b and Supplementary Fig. 1).

The *MEF2D* gene produces two alternatively spliced transcripts [17, 24] generating the ubiquitous α1 isoform and the inducible tissue-specific α2 isoform. Previously, the α2 transcript was detected only in muscle tissue and was studied in the murine myoblast cells C2C12 in more detail [17]. We detected low levels of the α2 isoform in human PBMC, as well as in THP-1, K562 and Daudi cell lines, which may suggest a putative role of the isoform in these cells. Interestingly, we found that the novel risk allele inhibits splicing of the α2 isoform transcribed from the minigene, while not affecting the α1 isoform.

The α2 isoform overexpressed in C2C12 cells regulated expression of a specific set of genes, only partially overlapping with those controlled by the α1 isoform [17]. Of note, two of the genes, specifically induced only by the α2 isoform, were *DNASE1L3* and *AIM2*, both previously shown to be involved in SLE pathogenesis [34–37]. The role of the α2 isoform in immune cells remains unclear at this stage, but in the light of our current results it certainly requires a comprehensive investigation. We do not know if the cell-specific enhancer with rs200395694 that presumably affects *MEF2D* expression could have an effect on α2 cell type-specific splicing, or if these two events are unrelated. For instance, in T cells, that do not express the α2 isoform, the main outcome of the risk allele would be on the regulation of gene expression. On the other hand, in monocytes normally producing the α2 isoform, the inhibition of it would have an effect on the pattern of the downstream target genes. It is tempting to speculate, for example, that direct correlation between the expression of the α2 transcript and *DNASE1L3* would result in suppression of *DNASE1L3* in monocytes in the risk genotype where the α2 is inhibited. The loss-of-function of *DNASE1L3* causes severe familial SLE [38].

In summary, in search for rare disease variants, usually missed in GWA studies, we used targeted re-sequencing of regulatory highly conserved regions in selected genes in a well-characterized cohort of SLE patients. We present evidence of genetic association of a rare variant rs200395694:G > T with SLE in Swedish patients. The risk allele is associated also with the triad of disease manifestations comprised of anti-Sm, anti-RNP antibodies, and Raynaud’s phenomenon. The SNP rs200395694:G > T is located in a cell type-specific enhancer and appears to influence expression and splicing of the *MEF2D* mRNA. While *MEF2D* is a widely expressed transcription factor, fine-tuning of its transcription and splicing in relevant cells may have an effect on downstream target genes and may contribute to the disease pathogenesis. We believe that our results support the omnigenic model for complex traits where *MEF2D* shows a rather modest association with SLE in our cohort of Swedish patients but apparent functional effect. Therefore, *MEF2D* could be considered as a peripheral gene for SLE.

## Electronic supplementary material

Supplementary tables

Supplementary methods

Supplementary figures
