# Supplementary material for: A rare regulatory variant in the MEF2D gene affects gene regulation and splicing and is associated with a SLE sub-phenotype in Swedish cohorts
Source: Eur J Hum Genet. 2018 Nov 20;27(3):432–41. doi: 10.1038/s41431-018-0297-x (PMC6460566; doi:10.1038/s41431-018-0297-x)
Supplement: Supplementary file 1 — Supplementary tables [file 41431_2018_297_MOESM1_ESM.docx]

**Supplementary Tables:**

**Supplementary Table 1.** Summary of basic clinical characteristics of SLE patients.

|  | **All patients (n= 156)** | **Swedish patients (n= 140)** |
| --- | --- | --- |
| Fulfill ACR criteria (1982), no. (%) | 156 (100) | 140 (100) |
| Fulfill ACR criteria (1997), no. (%) | 156 (100) | 140 (100) |
| Fulfill SLICC criteria (2012), no. (%) | 147 (94) | 132 (94) |
| Women, no. (%) | 139 (89) | 125 (89) |
| Men, no. (%) | 17 (11) | 15 (11) |
| Age at diagnosis, mean years (range) | 31 (9-78) | 31 (9-78) |
| Disease duration, mean years (range) | 16 (0-63) | 17 (0-63) |
| Total ACR criteria (1982), median (range) | 5 (4-9) | 6 (4-9) |
| SLICC DI, median (range) | 0 (0-6) | 1 (0-6) |

ACR= American College of Rheumatology; SLICC= Systemic Lupus International Collaborating Clinics; SLICC DI= SLICC Damage Index. Patients comprised of 144 (92%) of Scandinavian origin (140 Swedish, 3 Finnish, 1 Norwegian), four (2.6%) from other parts of Europe, seven (4.5%) of Asian origin, and one (0.6%) of South American origin as determined by country of birth from their medical records.

**Supplementary Table 2.** List of genes included in the array design: a total of 215 genes, comprising genes of the NFAT pathway (n=98), previously known SLE-associated genes and immune system-related genes (n=77) and genes in dog SLE candidate regions (n=40).

| **Gene** | **EntrezID (human)** | **Full name** |
| --- | --- | --- |
| *ACAN* | 176 | aggrecan |
| *AKT1* | 207 | v-akt murine thymoma viral oncogene homolog 1 |
| *ATF1* | 466 | activating transcription factor 1 |
| *AXL* | 558 | AXL receptor tyrosine kinase |
| *BCMA* | 608 | tumor necrosis factor receptor superfamily, member 17 |
| *BLK* | 640 | B lymphoid tyrosine kinase |
| *BTK* | 695 | Bruton agammaglobulinemia tyrosine kinase |
| *CASP3* | 836 | caspase 3, apoptosis-related cysteine peptidase |
| *CASP8* | 841 | caspase 8, apoptosis-related cysteine peptidase |
| *CASP10* | 843 | caspase 10, apoptosis-related cysteine peptidase |
| *RUNX1* | 861 | runt-related transcription factor 1 |
| *RUNX3* | 864 | runt-related transcription factor 3 |
| *CD3D* | 915 | CD3d molecule, delta (CD3-TCR complex) |
| *CD3E* | 916 | CD3e molecule, epsilon (CD3-TCR complex) |
| *CD4* | 920 | CD4 molecule |
| *CD27* | 939 | CD27 molecule |
| *CD28* | 940 | CD28 molecule |
| *CD80* | 941 | CD80 molecule |
| *CD86* | 942 | CD86 molecule |
| *CD36* | 948 | CD36 molecule (thrombospondin receptor) |
| *CD44* | 960 | CD44 molecule (Indian blood group) |
| *CD79A* | 973 | CD79a molecule, immunoglobulin-associated alpha |
| *CD79B* | 974 | CD79b molecule, immunoglobulin-associated beta |
| *CHUK* | 1147 | conserved helix-loop-helix ubiquitous kinase |
| *ATF2* | 1386 | activating transcription factor 2 |
| *CREM* | 1390 | cAMP responsive element modulator |
| *CTLA4* | 1493 | cytotoxic T-lymphocyte-associated protein 4 |
| *FCER1A* | 2205 | Fc fragment of IgE, high affinity I, receptor for; alpha polypeptide |
| *FCER1B* | 2206 | membrane-spanning 4-domains, subfamily A, member 2 (Fc fragment of IgE, high affinity I, receptor for; beta polypeptide) |
| *FCER1G* | 2207 | Fc fragment of IgE, high affinity I, receptor for; gamma polypeptide |
| *FCGR1A* | 2209 | Fc fragment of IgG, high affinity Ia, receptor (CD64) |
| *FCGR2A* | 2212 | Fc fragment of IgG, low affinity IIa, receptor (CD32) |
| *FOS* | 2353 | FBJ murine osteosarcoma viral oncogene homolog |
| *FRAP* | 2475 | mechanistic target of rapamycin (serine/threonine kinase) |
| *Fyn* | 2534 | FYN oncogene related to SRC, FGR, YES |
| *GAS6* | 2621 | growth arrest-specific 6 |
| *GATA3* | 2625 | GATA binding protein 3 |
| *GATA4* | 2626 | GATA binding protein 4 |
| *GRB2* | 2885 | growth factor receptor-bound protein 2 |
| *GSK3B* | 2932 | glycogen synthase kinase 3 beta |
| *H2AFZ* | 3015 | H2A histone family, member Z |
| *IRF8* | 3394 | interferon regulatory factor 8 |
| *IFNGR2* | 3460 | interferon gamma receptor 2 (interferon gamma transducer 1) |
| *IKBKB* | 3551 | inhibitor of kappa light polypeptide gene enhancer in B-cells, kinase beta |
| *IL2* | 3558 | interleukin 2 |
| *IL2RA* | 3559 | interleukin 2 receptor, alpha |
| *IL5* | 3567 | interleukin 5 (colony-stimulating factor, eosinophil) |
| *IL6* | 3569 | interleukin 6 (interferon, beta 2) |
| *IL6R* | 3570 | interleukin 6 receptor |
| *IL10* | 3586 | interleukin 10 |
| *IL12RB2* | 3595 | interleukin 12 receptor, beta 2 |
| *IL13* | 3596 | interleukin 13 |
| *IL17* | 3605 | interleukin 17A |
| *CXCL10* | 3627 | chemokine (C-X-C motif) ligand 10 |
| *IRAK1* | 3654 | interleukin-1 receptor-associated kinase 1 |
| *IRF1* | 3659 | interferon regulatory factor 1 |
| *IRF3* | 3661 | interferon regulatory factor 3 |
| *IRF4* | 3662 | interferon regulatory factor 4 |
| *IRF5* | 3663 | interferon regulatory factor 5 |
| *IRF7* | 3665 | interferon regulatory factor 7 |
| *ITGAL* | 3683 | integrin, alpha L (antigen CD11A (p180), lymphocyte function-associated antigen 1; alpha polypeptide) |
| *ITGAM* | 3684 | integrin, alpha M (complement component 3 receptor 3 subunit) |
| *ITGAX* | 3687 | integrin, alpha X (complement component 3 receptor 4 subunit) |
| *ITK* | 3702 | IL2-inducible T-cell kinase |
| *ITPR1* | 3708 | inositol 1,4,5-triphosphate receptor, type 1 |
| *JAK1* | 3716 | Janus kinase 1 |
| *JAK2* | 3717 | Janus kinase 2 |
| *JUN* | 3725 | jun proto-oncogene |
| *JUNB* | 3726 | jun B proto-oncogene |
| *JUND* | 3727 | jun D proto-oncogene |
| *KIR2DL3* | 3804 | killer cell immunoglobulin-like receptor, two domains, long cytoplasmic tail, 3 |
| *TNPO1* | 3842 | transportin 1 |
| *Lck* | 3932 | lymphocyte-specific protein tyrosine kinase |
| *LCP2* | 3937 | lymphocyte cytosolic protein 2 (SH2 domain containing leukocyte protein of 76kDa) |
| *LTA* | 4049 | lymphotoxin alpha (TNF superfamily, member 1) |
| *Lyn* | 4067 | v-yes-1 Yamaguchi sarcoma viral related oncogene homolog |
| *SMAD3* | 4088 | SMAD family member 3 |
| *MAF* | 4094 | v-maf musculoaponeurotic fibrosarcoma oncogene homolog (avian) |
| *MEF2D* | 4209 | myocyte enhancer factor 2D |
| *CIITA* | 4261 | class II, major histocompatibility complex, transactivator |
| *MTTP* | 4547 | microsomal triglyceride transfer protein |
| *MYD88* | 4615 | myeloid differentiation primary response gene (88) |
| *GADD45B* | 4616 | growth arrest and DNA-damage-inducible, beta |
| *NAIP* | 4671 | NLR family, apoptosis inhibitory protein |
| *NFKB1* | 4790 | nuclear factor of kappa light polypeptide gene enhancer in B-cells 1 |
| *NFKB2* | 4791 | nuclear factor of kappa light polypeptide gene enhancer in B-cells 2 (p49/p100) |
| *NFKBIA* | 4792 | nuclear factor of kappa light polypeptide gene enhancer in B-cells inhibitor, alpha |
| *NFKBIB* | 4793 | nuclear factor of kappa light polypeptide gene enhancer in B-cells inhibitor, beta |
| *PGAM1* | 5223 | phosphoglycerate mutase 1 (brain) |
| *PIK3CA* | 5290 | phosphoinositide-3-kinase, catalytic, alpha polypeptide |
| *PIK3CB* | 5291 | phosphoinositide-3-kinase, catalytic, beta polypeptide |
| *PIK3CD* | 5293 | phosphoinositide-3-kinase, catalytic, delta polypeptide |
| *PIK3CG* | 5294 | phosphoinositide-3-kinase, catalytic, gamma polypeptide |
| *PLCB2* | 5330 | phospholipase C, beta 2 |
| *PLCL1* | 5334 | phospholipase C-like 1 |
| *PLCG1* | 5335 | phospholipase C, gamma 1 |
| *PLCG2* | 5336 | phospholipase C, gamma 2 (phosphatidylinositol-specific) |
| *PON2* | 5445 | paraoxonase 2 |
| *PPARG* | 5468 | peroxisome proliferator-activated receptor gamma |
| *PPP2CA* | 5515 | protein phosphatase 2, catalytic subunit, alpha isozyme |
| *PPP3CA* | 5530 | protein phosphatase 3, catalytic subunit, alpha isozyme |
| *PRKCQ* | 5588 | protein kinase C, theta |
| *MAPK3* | 5595 | mitogen-activated protein kinase 3 |
| *PROS1* | 5627 | protein S (alpha) |
| *PTPN3* | 5774 | protein tyrosine phosphatase, non-receptor type 3 |
| *PTPRM* | 5797 | protein tyrosine phosphatase, receptor type, M |
| *RAF1* | 5894 | v-raf-1 murine leukemia viral oncogene homolog 1 |
| *RELA* | 5970 | v-rel reticuloendotheliosis viral oncogene homolog A (avian) |
| *RELB* | 5971 | v-rel reticuloendotheliosis viral oncogene homolog B |
| *RNAseL* | 6041 | ribonuclease L (2',5'-oligoisoadenylate synthetase-dependent) |
| *RORA* | 6095 | RAR-related orphan receptor A |
| *RORC* | 6097 | RAR-related orphan receptor C |
| *RPS17* | 6218 | ribosomal protein S17 |
| *SOS1* | 6654 | son of sevenless homolog 1 (Drosophila) |
| *OPN* | 6696 | secreted phosphoprotein 1 |
| *STAT1* | 6772 | signal transducer and activator of transcription 1, 91kDa |
| *STAT2* | 6773 | signal transducer and activator of transcription 2, 113kDa |
| *STAT3* | 6774 | signal transducer and activator of transcription 3 (acute-phase response factor) |
| *STAT4* | 6775 | signal transducer and activator of transcription 4 |
| *STAT6* | 6778 | signal transducer and activator of transcription 6, interleukin-4 induced |
| *ABCC8* | 6833 | ATP-binding cassette, sub-family C (CFTR/MRP), member 8 |
| *SYK* | 6850 | spleen tyrosine kinase |
| *TCRA* | 6955 | T cell receptor alpha locus |
| *TCRB* | 6957 | T cell receptor beta locus |
| *TRAF3* | 7187 | TNF receptor-associated factor 3 |
| *TRAF6* | 7189 | TNF receptor-associated factor 6 |
| *TWIST1* | 7291 | twist homolog 1 (Drosophila) |
| *TNFSF4* | 7292 | tumor necrosis factor (ligand) superfamily, member 4 |
| *TNFRSF4* | 7293 | tumor necrosis factor receptor superfamily, member 4 |
| *TXN* | 7295 | thioredoxin |
| *TYK2* | 7297 | tyrosine kinase 2 |
| *TYRO3* | 7301 | TYRO3 protein tyrosine kinase |
| *VRK1* | 7443 | vaccinia related kinase 1 |
| *XPO1* | 7514 | exportin 1 (CRM1 homolog, yeast) |
| *ZAP70* | 7535 | zeta-chain (TCR) associated protein kinase 70kDa |
| *AP3B2* | 8120 | adaptor-related protein complex 3, beta 2 subunit |
| *PLA2G6* | 8398 | phospholipase A2, group VI (cytosolic, calcium-independent) |
| *MAPKSP1* | 8649 | MAPK scaffold protein 1 |
| *SOCS1* | 8651 | suppressor of cytokine signaling 1 |
| *TRADD* | 8717 | TNFRSF1A-associated via death domain |
| *RIPK1* | 8737 | receptor (TNFRSF)-interacting serine-threonine kinase 1 |
| *RIP2* | 8767 | receptor-interacting serine-threonine kinase 2 |
| *FADD* | 8772 | Fas (TNFRSF6)-associated via death domain |
| *TNFRSF18* | 8784 | tumor necrosis factor receptor superfamily, member 18 |
| *SOCS3* | 9021 | suppressor of cytokine signaling 3 |
| *MAPKAPK2* | 9261 | mitogen-activated protein kinase-activated protein kinase 2 |
| *SOCS6* | 9306 | suppressor of cytokine signaling 6 |
| *HOMER2* | 9455 | homer homolog 2 (Drosophila) |
| *AKAP5* | 9495 | A kinase (PRKA) anchor protein 5 |
| *IKBKE* | 9641 | inhibitor of kappa light polypeptide gene enhancer in B-cells, kinase epsilon |
| *TBKBP1* | 9755 | TBK1 binding protein 1 |
| *SNAP91* | 9892 | synaptosomal-associated protein, 91kDa homolog (mouse) |
| *TANK* | 10010 | TRAF family member-associated NFKB activator |
| *TNIP1* | 10318 | TNFAIP3 interacting protein 1 |
| *IRF9* | 10379 | interferon regulatory factor 9 |
| *MERTK* | 10461 | c-mer proto-oncogene tyrosine kinase |
| *CD226* | 10666 | CD226 molecule |
| *BAFF* | 10673 | tumor necrosis factor (ligand) superfamily, member 13b |
| *MAP3K2* | 10746 | mitogen-activated protein kinase kinase kinase 2 |
| *GADD45G* | 10912 | growth arrest and DNA-damage-inducible, gamma |
| *TRAM1* | 23471 | translocation associated membrane protein 1 |
| *CABIN1* | 23523 | calcineurin binding protein 1 |
| *DDX58* | 23586 | DEAD (Asp-Glu-Ala-Asp) box polypeptide 58 |
| *PTPN22* | 26191 | protein tyrosine phosphatase, non-receptor type 22 (lymphoid) |
| *ANKRD2* | 26287 | ankyrin repeat domain 2 (stretch responsive muscle) |
| *LAT* | 27040 | linker for activation of T cells |
| *DAPP1* | 27071 | dual adaptor of phosphotyrosine and 3-phosphoinositides |
| *TBK1* | 29110 | TANK-binding kinase 1 |
| *BLNK* | 29760 | B-cell linker |
| *TBX21* | 30009 | T-box 21 |
| *IL22* | 50616 | interleukin 22 |
| *FOXP3* | 50943 | forkhead box P3 |
| *EXOSC1* | 51013 | exosome component 1 |
| *IRAK4* | 51135 | interleukin-1 receptor-associated kinase 4 |
| *TLR7* | 51284 | toll-like receptor 7 |
| *TLR8* | 51311 | toll-like receptor 8 |
| *EMCN* | 51705 | endomucin |
| *TLR9* | 54106 | toll-like receptor 9 |
| *EPB41L4B* | 54566 | erythrocyte membrane protein band 4.1 like 4B |
| *BANK1* | 55024 | B-cell scaffold protein with ankyrin repeats 1 |
| *BATF3* | 55509 | basic leucine zipper transcription factor, ATF-like 3 |
| *MAVS* | 57506 | mitochondrial antiviral signaling protein |
| *SLAMF7* | 57823 | SLAM family member 7 |
| *IL21* | 59067 | interleukin 21 |
| *HAPLN2* | 60484 | hyaluronan and proteoglycan link protein 2 |
| *IFIH1* | 64135 | interferon induced with helicase C domain 1 |
| *MMS19* | 64210 | MMS19 nucleotide excision repair homolog (S. cerevisiae) |
| *NAP1* | 64343 | 5-azacytidine induced 2 |
| *BCL11B* | 64919 | B-cell CLL/lymphoma 11B (zinc finger protein) |
| *DHX58* | 79132 | DEXH (Asp-Glu-X-His) box polypeptide 58 |
| *DNAJB14* | 79982 | DnaJ (Hsp40) homolog, subfamily B, member 14 |
| *UBTD1* | 80019 | ubiquitin domain containing 1 |
| *ZBP1* | 81030 | Z-DNA binding protein 1 |
| *ACSBG2* | 81616 | acyl-CoA synthetase bubblegum family member 2 |
| *ZDHHC16* | 84287 | zinc finger, DHHC-type containing 16 |
| *MINA* | 84864 | MYC induced nuclear antigen |
| *ORAI1* | 84876 | ORAI calcium release-activated calcium modulator 1 |
| *HOGA1* | 112817 | 4-hydroxy-2-oxoglutarate aldolase 1 |
| *TIRAP* | 114609 | toll-interleukin 1 receptor (TIR) domain containing adaptor protein |
| *DDIT4L* | 115265 | DNA-damage-inducible transcript 4-like |
| *BAFFR* | 115650 | tumor necrosis factor receptor superfamily, member 13C |
| *DNTTIP1* | 116092 | deoxynucleotidyltransferase, terminal, interacting protein 1 |
| *WHAMM* | 123720 | WAS protein homolog associated with actin, golgi membranes and microtubules |
| *FSD2* | 123722 | fibronectin type III and SPRY domain containing 2 |
| *WFDC3* | 140686 | WAP four-disulfide core domain 3 |
| *HAPLN3* | 145864 | hyaluronan and proteoglycan link protein 3 |
| *TRIF* | 148022 | toll-like receptor adaptor molecule 1 |
| *WFDC13* | 164237 | WAP four-disulfide core domain 13 |
| *WFDC11* | 259239 | WAP four-disulfide core domain 11 |
| *WFDC10B* | 280664 | WAP four-disulfide core domain 10B |
| *SPINT4* | 391253 | serine peptidase inhibitor, Kunitz type 4 |
| *C10orf62* | 414157 | chromosome 10 open reading frame 62 |
| *PGAM4* | 441531 | phosphoglycerate mutase family member 4 |
| *SCARNA15* | 677778 | small Cajal body-specific RNA 15 |
| *hsa-MIR-1255A* | 100302193 | microRNA 1255a |

**Supplementary Table 3.** Descriptive statistics showing amount of data, mapped reads and coverage calculations for each pool sequenced.

| **Pool** | **Individuals** | **Number of reads (Gb)** | **Mapped reads (%)** | **Reads on tiled (%)** | **Average Coverage (X)** | **Coverage (0 X)** | **Coverage (1-100 X)** | **Coverage (>100 X)** |
| --- | --- | --- | --- | --- | --- | --- | --- | --- |
| 1 | 11 | 40.3 | 92.9 | 39.8 | 2782 | 0.1 | 0.6 | 99.3 |
| 2 | 17 | 33.6 | 97.4 | 49.7 | 2901 | 0.3 | 1.1 | 98.6 |
| 3 | 15 | 40.3 | 98.3 | 57.5 | 4026 | 0.1 | 0.5 | 99.3 |
| 4 | 17 | 32.0 | 98.2 | 65.7 | 3655 | 0.4 | 1.0 | 98.7 |
| 5 | 17 | 41.1 | 98.3 | 61.6 | 4396 | 0.7 | 1.5 | 97.8 |
| 6 | 15 | 41.5 | 98.4 | 60.9 | 4386 | 0.2 | 0.7 | 99.1 |
| 7 | 17 | 39.7 | 98.4 | 63.3 | 4362 | 0.8 | 1.6 | 97.6 |
| 8 | 15 | 40.9 | 97.7 | 65.6 | 4658 | 0.1 | 0.5 | 99.3 |
| 9 | 16 | 43.7 | 97.6 | 63.3 | 4801 | 0.5 | 1.2 | 98.3 |
| Controls | 17 | 23.1 | 79.6 | 44.4 | 1780 | 0.0 | 0.9 | 99.1 |

**Supplementary Table 4.** Position, alleles, type, and identifier of the 10 SNPs selected.

| Position* | Ref | Alt | Genes | SNP type | Rs# or SS# |
| --- | --- | --- | --- | --- | --- |
| chr1:156450591 | G | T | *MEF2D* | intron | rs200395694 |
| chr1:25253141 | T | C | *RUNX3* | intron | rs181816122 |
| chr1:25349498 | C | T | *RUNX3* | upstream | rs114903524 |
| chr7:128581094 | A | G | *IRF5* | intron | rs150639578 |
| chr14:22958952 | G | A | *TCRA* | intron | rs867059436 |
| chr14:23025555 | C | T | *TCRA* | upstream | rs561936568 |
| chr15:89437973 | G | A | *HAPLN3* | intron | rs576275580 |
| chr21:36169429 | G | A | *RUNX1* | intron | rs551929460 |
| chr21:36258521 | C | G | *RUNX1* | intron | ss2019492549 |
| chr21:36259347 | G | A | *RUNX1* | synonymous | rs147889692 |

*Human genome 19 (GRCh37/hg19)

**Supplementary Table 5.** Association between 3 candidate SNPs and SLE.

| Rs# or SS# | Position* | Ref | Alt | Genes | P-value |
| --- | --- | --- | --- | --- | --- |
| rs200395694 | chr1:156450591 | G | T | *MEF2D* | 0.014 |
| rs867059436 | chr14:22958952 | G | A | *TCRA* | 0.188 |
| rs576275580 | chr15:89437973 | G | A | *HAPLN3* | 0.818 |

*Human genome 19 (GRCh37/hg19)

**Supplementary Table 6.** Transcription factors with largest differences in binding affinities for *MEF2D* rs200395694 alleles.

| Rank | Difference log(p) | Ref-C p-value | Alt-A p-value | Transcription factor |
| --- | --- | --- | --- | --- |
| 1 | -3.38 | 1.71e-07 | <7.22e-11 | GABP |
| 2 | -1.45 | 0.383 | 0.0136 | P300 |
| 3 | -1.33 | 0.222 | 0.0103 | AP2REP |
| 4 | -1.23 | 0.145 | 0.0085 | ELK1 |
| 5 | -1.2 | 0.198 | 0.0125 | HIF1 |
| 6 | -1.09 | 0.129 | 0.0105 | HIF1 |
| 7 | -1.06 | 0.044 | 0.0038 | TEL2 |
| 8 | -0.97 | 0.0041 | 0.00044 | E74A |
| 9 | -0.97 | 0.0806 | 0.0086 | HEN1 |
| 10 | -0.96 | 0.169 | 0.0187 | PEA3 |
| 11 | -0.86 | 0.352 | 0.0484 | CETS1P54 |
| 12 | -0.84 | 0.076 | 0.0109 | NKX25 |
| 13 | 0.80 | 0.0298 | 0.19 | ZIC1 |
| 14 | 0.78 | 0.00378 | 0.0231 | MARZ |
| 15 | -0.78 | 0.0169 | 0.00279 | ESTS1 |

The transcription factor database used was TRANSFAC and p-values were calculated with sTRAP.
