# Supplementary material for: A rare regulatory variant in the MEF2D gene affects gene regulation and splicing and is associated with a SLE sub-phenotype in Swedish cohorts
Source: Eur J Hum Genet. 2018 Nov 20;27(3):432–41. doi: 10.1038/s41431-018-0297-x (PMC6460566; doi:10.1038/s41431-018-0297-x)
Supplement: Supplementary file 2 — Supplementary methods [file 41431_2018_297_MOESM2_ESM.pdf]

**Supplementary Methods:**

**Gene array capture and sequence analysis**

For all 215 genes chosen (**Supplementary Table 2**) for targeted re-sequencing the following regions were included in the sequence analysis: all annotated coding exons (±20 bp from exon/intron boundaries), 5´ UTRs (±2 kb), 3' UTRs (+20 bp) and all conserved elements based on 29 mammals alignment with a SiPhy [1] lodscore of >7.5, located within 100 kb 5' and 3' of the genes (hg18/NCBI Build 36.1). Each conserved element was extended to a minimum of 100 bp. A total of 2.9 Mb of coding regions and 2.7 Mb of conserved elements were included. The tiling array comprised a total of 5,059,619 bp in total. Sequencing libraries of the targeted region were prepared using 2 μg of genomic DNA from each individual with NimbleGen EZ Choice custom array (Roche NimbleGen). DNA was sonicated into ~ 350 bp fragments, and ligated with Illumina Paired-End adapters. DNA samples from the 156 patients were allocated into ten different pools (average 15 individuals per pool), and DNA from 17 healthy controls was combined into one pool. All pools were hybridized to the SeqCap EZ array, after which they were PCR amplified (18 cycles). Capture efficiency was measured using real-time quantitative PCR with Fast SYBR green master mix and the ABI7900HT sequence Detection System (Applied Biosystems, Carlsbad, CA) according to manufacturer's protocol. An enrichment factor of >200x was considered to be successful when compared with un-captured samples. All but one patient pool passed the quality check. Sequencing was performed using Illumina HighSeq2000 paired-end sequencing at the SNP&SEQ Technology Platform (National Genomics infrastructure, SciLifeLab, Uppsala, Sweden), yielding 100 bp reads.

Sequence alignment to the human genome 18 (NCBI Build 36.1) was

completed with the Burrows-Wheeler Alignment tool (BWA) [2]. Data alignment was inspected with Samtools software package [3] and realignment of data was performed using the Genome Analysis Toolkit (GATK) [4]. Only reads with Phred quality scores >20 were included for further analyses. Depth and breadth of coverage for our selected target regions was calculated with BedTools [5] with the coverageBed command. SNP and indel calling was performed with an in-house Perl script to create vcf files. PCR duplicates were included for further analysis, since non-barcoded individuals were used in the same pool and to avoid calculating allele frequencies incorrectly. Given the high coverage 3,775 (3,774 ± 935) and the number of individuals in the different pools, a 1% cut-off was set as being inclusive for SNP calls, for each pool. This meant that a SNP needed to have at least 10 X coverage to be further included in the analysis. The vcf files were imported to seqscoring (www.seqscoring.org) [6] for comparative analyses between case and control pools. Population allele frequencies for all SNPs were compared to the 1000 genomes database (1000G; 12/17/2012)[7] and European allele frequencies were extracted where available. The data was compared to dbSNP (dbSNP137)[8] in Galaxy [9] for identification of novel SNPs.

Allelic variant enrichment was assessed first by assigning “case-only variants” (variants found only in cases and not in controls of 1000 genomes or dbSNP137) to genes, which were considered to be enriched when comprising five or more variants. The gene regions were defined by GREAT[10] based on the human genome 18 (NCBI Build 36.1). The variants from the enriched genes were lifted to the human genome 19 (GRCh37/hg19) and evaluated for potential function, e.g. exonic variants for their effects on protein as predicted with SIFT [11]. For non-coding variants any potential regulatory function was determined using ENCODE data, including histone

modifications, DNase I hypersensitivity clusters and chromatin immunoprecipitation sequencing (ChIP-seq) data. Additionally, a PhyloP score, an index based on the alignment of DNA sequences from 100 vertebrates[12], was assigned to each SNP in order to assess the evolutionary conservation, which may serve as an indicator of potential function. The PhyloP score assigns positive values for conserved bases (slower than expected evolution) and negative values for fast evolving bases, the genome score range is from -20 to 9. Variants were counted as functional candidates when fitting at least three of the following criteria: overlapping with promoter or enhancer associated histone modifications, DNase I hypersensitive site, ChIP-seq peaks or positive PhyloP score.

**Electrophoretic mobility shift assay**

Electrophoretic mobility shift assays (EMSA) were performed using LightShift Chemiluminescent EMSA kit (Thermo Scientific), according to the manufacturer's protocol. Probes of 31 bp were designed for the reference and alternative alleles; forward probes were biotin labeled at the 5' end (Ref-for 5'-GGAAGGGGCCGGAAGGGGGGGCCAACAGAGA-3', Ref-rev 5'-TCTCTGTTGGCCCCCCCTTCCGGCCCCTTCC-3'; Alt-for 5'-GGAAGGGGCCGGAAGTGGGGGCCAACAGAGA-3', Alt-rev 5'-TCTCTGTTGGCCCCCACTTCCGGCCCCTTCC-3'). Probes were annealed (40 fmol) and incubated with nuclear extracts (~10-20 μg) in a binding reaction containing: 1X LightShift Chemiluminescent EMSA binding buffer, 1μg of poly(dI.dC), 7.5% glycerol, 0.0063% NP-40, 30.1mM KCl, 2mM $MgCl_2$, 0.1 mM EDTA. The reaction was incubated on ice for 40 minutes and run on a 5% TBE Criterion gel (Bio-Rad). Competition was performed with unlabeled probe at 100 X

molar excess. Image Lab software (Bio-Rad) was used to visualize and capture images. Nuclear protein extracts were prepared with NucBuster kit (Novagen) from Jurkat and Daudi cell lines. In addition, an aliquot of Jurkat cells were stimulated with 20 ng/ml phorbol myristyl acetate (PMA) and 20 ng/ml ionomycin for 12 hours.

### Luciferase reporter assay

DNA fragment of 176 bp for the selected candidate variant rs200395694 was amplified by PCR using genomic DNA obtained from individuals with known genotypes. The following primers were used for amplification: For-5'-ACTCACTCTGTCTCTGTTGGCCCCC-3', Rev-5'-CTATAACTCTGCATCATTTTATCA-3'. PCR products were cloned in the pGL4.26 reporter vector (Promega). After sequence validation, plasmids were purified with EndoFree Plasmid Maxi Kit (Qiagen) and transfected into cultured cells. The allelic constructs were tested in Jurkat, Daudi, K562, THP-1 and HeLa cells. Transfections were performed using Lipofectamine 2000 (Invitrogen) according to the manufacturer's protocol. Thirty-six hours after transfection, an aliquot of Jurkat cells was additionally stimulated for 12 hours (as described for EMSA), then harvested and assayed for the Firefly and Renilla luciferase activities with the Dual-Luciferase Reporter Assay System (Promega). The experiment was repeated three times with four technical replicates for each plasmid. The un-paired Student's t-test was used for statistical analysis of the differences in allele-specific effects on transcriptional activity.

### Minigene assay

The minigenes were transfected into Jurkat, HEK293 and THP-1using Lipofectamine 2000 (Invitrogen). C2C12 cells were transfected with jetPRIME (Polyplus Transfection) according to the manufacturer's protocol. Jurkat cells were additionally stimulated with 20 ng/ml of PMA and 0.5 μM ionomycin for 12 hours before collecting cells, THP-1 cells were stimulated with 100 ng/ml of LPS and 10ng/ml of interferon γ. The transfection was stopped after 48 hours by adding Trizol reagent (Invitrogen). The medium for C2C12 cells was changed 8 hrs posttransfection, half of the samples were incubated in the medium with 10% of fetal bovine serum, another half – in the medium with 2% horse serum to induce cell differentiation. The total incubation time of C2C12 cells was 72 hours. All transfection experiments were repeated four to five times.

Total RNA was extracted either from purified PBMC of healthy donors, cultured cells from different cell lines or from transfected cells using Trizol and treated with RQ1 DNase I (Promega). cDNA synthesis was performed using 1μg of DNase-treated RNA in a buffer containing 1 unit of MuLV reverse transcriptase (ThermoFisher), oligo(dT) primers, 1 mM dNTPs and RNase inhibitor (ThermoFisher). The quantitative real-time PCR was performed using SYBR green for detection. The expression levels of two isoforms with alternative exons α1 and α2 generated from the minigenes were normalized to the levels of the neomycin gene expressed from the same plasmid using the comparative $2^{-\Delta\Delta Ct}$ method. The following forward primers were used for detection of the α1 isoform 5'-GACATCATCGAGACCCTGAGGA-3' and the α2 isoform 5'-GACATCATCGAGGCGCTGCAC-3', and a common reverse primer 5'-GGTTAGGGATAGGCTTACCTTCGA-3'; the primers for neomycin transcript were 5'-TGGCGGACCGCTATCAGGACATA-3' and 5'-

ACCCCAGAGTCCCGCTCAGAAG-3'. Statistical analysis was performed using an unpaired 2-tailed t-test with GraphPad software (http://www.graphpad.com). The detection of endogenous transcripts was performed by PCR with forward isoform-specific primers and a common reverse primer 5'-GGCATTGTTCAAGTGATGCATT-3'.

**References:**


1. Garber M, Guttman M, Clamp M, Zody MC, Friedman N, Xie X. Identifying novel constrained elements by exploiting biased substitution patterns. Bioinformatics. 2009;25(12):i54-62.
2. Li H, Durbin R. Fast and accurate short read alignment with Burrows-Wheeler transform. Bioinformatics. 2009;25(14):1754-60.
3. Li H, Handsaker B, Wysoker A, Fennell T, Ruan J, Homer N, et al. The Sequence Alignment/Map format and SAMtools. Bioinformatics. 2009;25(16):2078-9.
4. DePristo MA, Banks E, Poplin R, Garimella KV, Maguire JR, Hartl C, et al. A framework for variation discovery and genotyping using next-generation DNA sequencing data. Nature genetics. 2011;43(5):491-8.
5. Quinlan AR, Hall IM. BEDTools: a flexible suite of utilities for comparing genomic features. Bioinformatics. 2010;26(6):841-2.
6. Truve K, Eriksson O, Norling M, Wilbe M, Mauceli E, Lindblad-Toh K, et al. SEQscoring: a tool to facilitate the interpretation of data generated with next generation sequencing technologies. EMBnet journal. 2011;17(1):38-45.
7. Genomes Project C, Abecasis GR, Auton A, Brooks LD, DePristo MA, Durbin RM, et al. An integrated map of genetic variation from 1,092 human genomes. Nature. 2012;491(7422):56-65.
8. Sherry ST, Ward MH, Kholodov M, Baker J, Phan L, Smigielski EM, et al. dbSNP: the NCBI database of genetic variation. Nucleic acids research. 2001;29(1):308-11.
9. Blankenberg D, Von Kuster G, Coraor N, Ananda G, Lazarus R, Mangan M, et al. Galaxy: a web-based genome analysis tool for experimentalists. Curr Protoc Mol Biol. 2010;Chapter 19:Unit 19 0 1-21.
10. McLean CY, Bristor D, Hiller M, Clarke SL, Schaar BT, Lowe CB, et al. GREAT improves functional interpretation of cis-regulatory regions. Nat Biotechnol. 2010;28(5):495-501.
11. Ng PC, Henikoff S. SIFT: Predicting amino acid changes that affect protein function. Nucleic acids research. 2003;31(13):3812-4.
12. Siepel A, Haussler D. Combining phylogenetic and hidden Markov models in biosequence analysis. Journal of computational biology : a journal of computational molecular cell biology. 2004;11(2-3):413-28.
