# Supplementary material for: A rare regulatory variant in the MEF2D gene affects gene regulation and splicing and is associated with a SLE sub-phenotype in Swedish cohorts
Source: Eur J Hum Genet. 2018 Nov 20;27(3):432–41. doi: 10.1038/s41431-018-0297-x (PMC6460566; doi:10.1038/s41431-018-0297-x)

Supplementary Figures

Supplementary Figure 1: The regulatory potential of SNP rs200395694 was assessed by luciferase reporter assay in different cell lines. Bars represent mean values ±SD. RLU, relative light units. Statistical analysis was done using an unpaired t test.

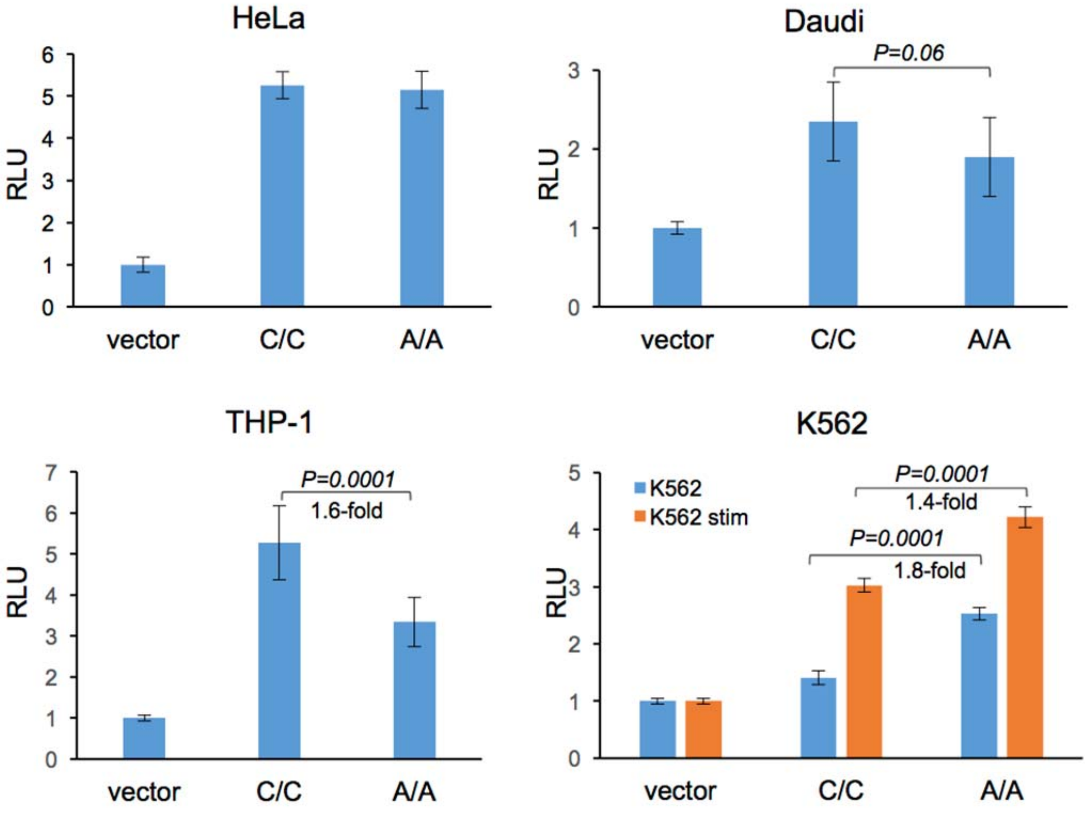

Supplementary Figure 2: Expression of *MEF2D* isoforms in different cells. M-100 bp DNA ladder, NTC-no template control. C2C12 stimulation was performed for 3 days with 2% horse serum. In case of mouse C2C12, the annealing temperature was lowered from 64°C to 58°C to allow amplification with human-specific primers.

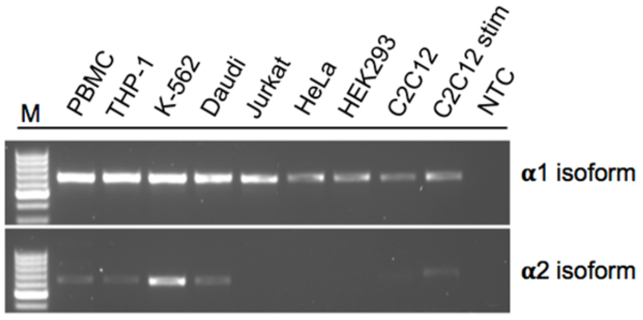

Supplementary Figure 3: Expression of *MEF2D* isoforms from minigenes transfected in Jurkat and HEK293 cells. Jurkat cells were stimulated with PMA and ionomycin for 12 hours. Bars represent mean values ±SEM. Statistical analysis was done using an unpaired t test.

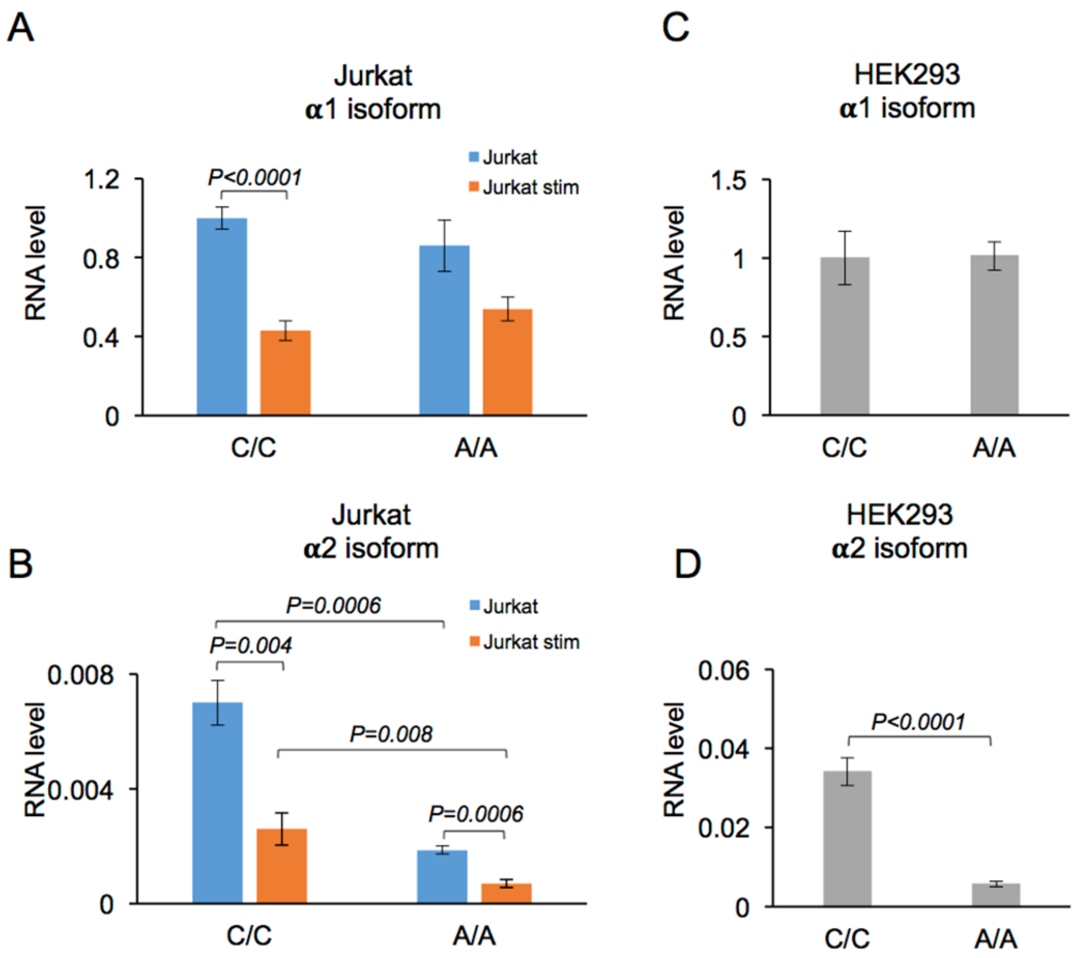

Supplement: Supplementary file 3 — Supplementary figures [file 41431_2018_297_MOESM3_ESM.pdf]
